# Assessment of gene expression of intracellular calcium channels, pumps and exchangers with epidermal growth factor-induced epithelial-mesenchymal transition in a breast cancer cell line

**DOI:** 10.1186/1475-2867-13-76

**Published:** 2013-07-29

**Authors:** Felicity M Davis, Michelle T Parsonage, Peter J Cabot, Marie-Odile Parat, Erik W Thompson, Sarah J Roberts-Thomson, Gregory R Monteith

**Affiliations:** 1 School of Pharmacy, The University of Queensland, Brisbane, QLD 4072, Australia; 2 St Vincent’s Institute, Fitzroy, VIC 3065, Australia; 3 Department of Surgery, St. Vincent’s Hospital, University of Melbourne, Fitzroy, VIC 3065, Australia

**Keywords:** Breast cancer calcium, EMT, IP_3_R, RYR, SERCA, SPCA, MCU, NCLX

## Abstract

**Background:**

Epithelial-mesenchymal transition (EMT) is a process implicated in cancer metastasis that involves the conversion of epithelial cells to a more mesenchymal and invasive cell phenotype. In breast cancer cells EMT is associated with altered store-operated calcium influx and changes in calcium signalling mediated by activation of cell surface purinergic receptors. In this study, we investigated whether MDA-MB-468 breast cancer cells induced to undergo EMT exhibit changes in mRNA levels of calcium channels, pumps and exchangers located on intracellular calcium storing organelles, including the Golgi, mitochondria and endoplasmic reticulum (ER).

**Methods:**

Epidermal growth factor (EGF) was used to induce EMT in MDA-MB-468 breast cancer cells. Serum-deprived cells were treated with EGF (50 ng/mL) for 12 h and gene expression was assessed using quantitative RT-PCR.

**Results and conclusions:**

These data reveal no significant alterations in mRNA levels of the Golgi calcium pump secretory pathway calcium ATPases (SPCA1 and SPCA2), or the mitochondrial calcium uniporter (MCU) or Na^+^/Ca^2+^ exchanger (NCLX). However, EGF-induced EMT was associated with significant alterations in mRNA levels of specific ER calcium channels and pumps, including (sarco)-endoplasmic reticulum calcium ATPases (SERCAs), and inositol 1,4,5-trisphosphate receptor (IP_3_R) and ryanodine receptor (RYR) calcium channel isoforms. The most prominent change in gene expression between the epithelial and mesenchymal-like states was RYR2, which was enriched 45-fold in EGF-treated MDA-MB-468 cells. These findings indicate that EGF-induced EMT in breast cancer cells may be associated with major alterations in ER calcium homeostasis.

## Background

EMT facilitates cancer cell invasion and metastasis formation, and has also been linked to the acquisition of a stem cell-like phenotype, anchorage-independent growth and chemoresistance in cancer cell lines and clinical samples [1]–[4]. The phenotypic changes associated with EGF-induced EMT are well characterised in the human breast cancer cell line MDA-MB-468, and include changes in cell morphology; increased expression of the transcription factor Twist and the intermediate filament protein vimentin; and reduced E-cadherin expression following chronic EGF treatment [5]–[8]. In addition, EGF-induced EMT in MDA-MB-468 breast cancer cells has been linked to altered plasma membrane calcium influx [6,7,9].

Increases in cytosolic calcium may occur via calcium entry through calcium permeable plasma membrane channels, including transient receptor potential (TRP), voltage-gated and store-operated calcium channels, or due to the release of calcium from internal stores [10]–[12]. Calcium signals arising via influx or store-release have distinct spatiotemporal profiles, modes of activation, and cellular responses [13]. These two processes often occur in parallel, for example calcium store release triggering store-operated calcium influx [14], and calcium influx via specific plasma membrane calcium channels potentially causing calcium release from the ER via calcium-induced calcium release (CICR) [15]. Mitochondrial calcium buffering may also alter the nature of cytosolic calcium responses [16]–[19]. Thus changes in the expression of calcium channels, pumps and exchangers located on intracellular calcium storing organelles may alter the nature of the cytosolic calcium response to a range of stimuli. However, while altered store-operated [6,9] and non-stimulated [6] plasmalemmal calcium influx have been linked to the acquisition of a more mesenchymal phenotype in breast cancer cells, changes in calcium channels, pumps and exchangers located on intracellular calcium storing organelles have not been assessed during this process.

A phenotypic conversion and cellular heterogeneity comparable to EMT, which is associated with a remodelling of intracellular calcium homeostasis, is observed in vascular smooth muscle cells [20]. In response to cues from the microenvironment, vascular smooth muscle cells can switch from a contractile to a synthetic (proliferative) phenotype [20]. Vascular smooth muscle cell switching is associated with changes in the expression of ER calcium channels and pumps, including reduced expression of RYR3 and SERCA2a [20,21]. Vascular smooth muscle cells are therefore one example of how intracellular calcium channels and pumps may be remodelled to meet the cellular requirements of specific phenotypes.

In this study we assessed whether alterations in mRNA levels of Golgi, mitochondrial and ER calcium channels, pumps and exchangers were associated with EGF-induced EMT in MDA-MB-468 breast cancer cells.

## Results and discussion

### EGF-induced EMT in MDA-MB-468 cells

**Figure 1:**
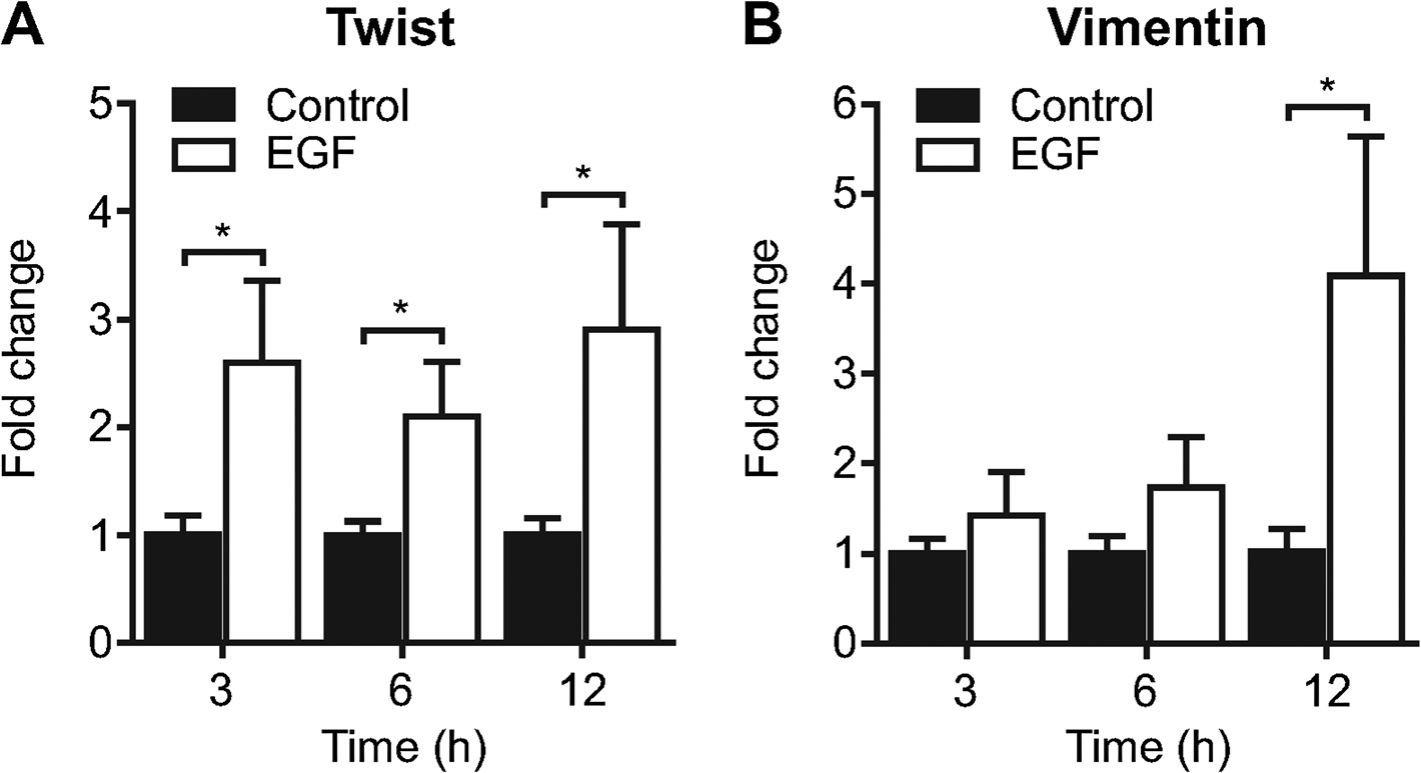
**EGF-induced EMT in MDA-MB-468 breast cancer cells.** MDA-MB-468 breast cancer cells were serum starved and treated with EGF (50 ng/mL) as indicated to induce EMT. **A)** Twist and **B)** vimentin mRNA expression were assessed using real time RT-PCR at 3, 6 and 12 h following EGF treatment. Bar graphs show mean ± S.D. for nine individual wells from three independent experiments. The effect of EGF at each time point was assessed using two-way ANOVA with Bonferroni’s multiple comparisons post-tests. * *P* < 0.05.

MDA-MB-468 breast cancer cells treated with EGF (50 ng/mL) for 3, 6 or 12 h showed a significant increase in mRNA levels of Twist (Figure 1A), a nuclear transcription factor and marker of EMT [22]. This early EMT event was followed by an increase in vimentin mRNA at 12 h post-EGF treatment (Figure 1B). These findings were consistent with previous studies using this model of EGF-induced EMT [5]–[8]. We assessed gene expression of calcium channels, pumps and exchangers located on the Golgi, mitochondria and ER with EMT after treatment with EGF for 12 h.

### Golgi: SPCA1 and SPCA2

**Figure 2:**
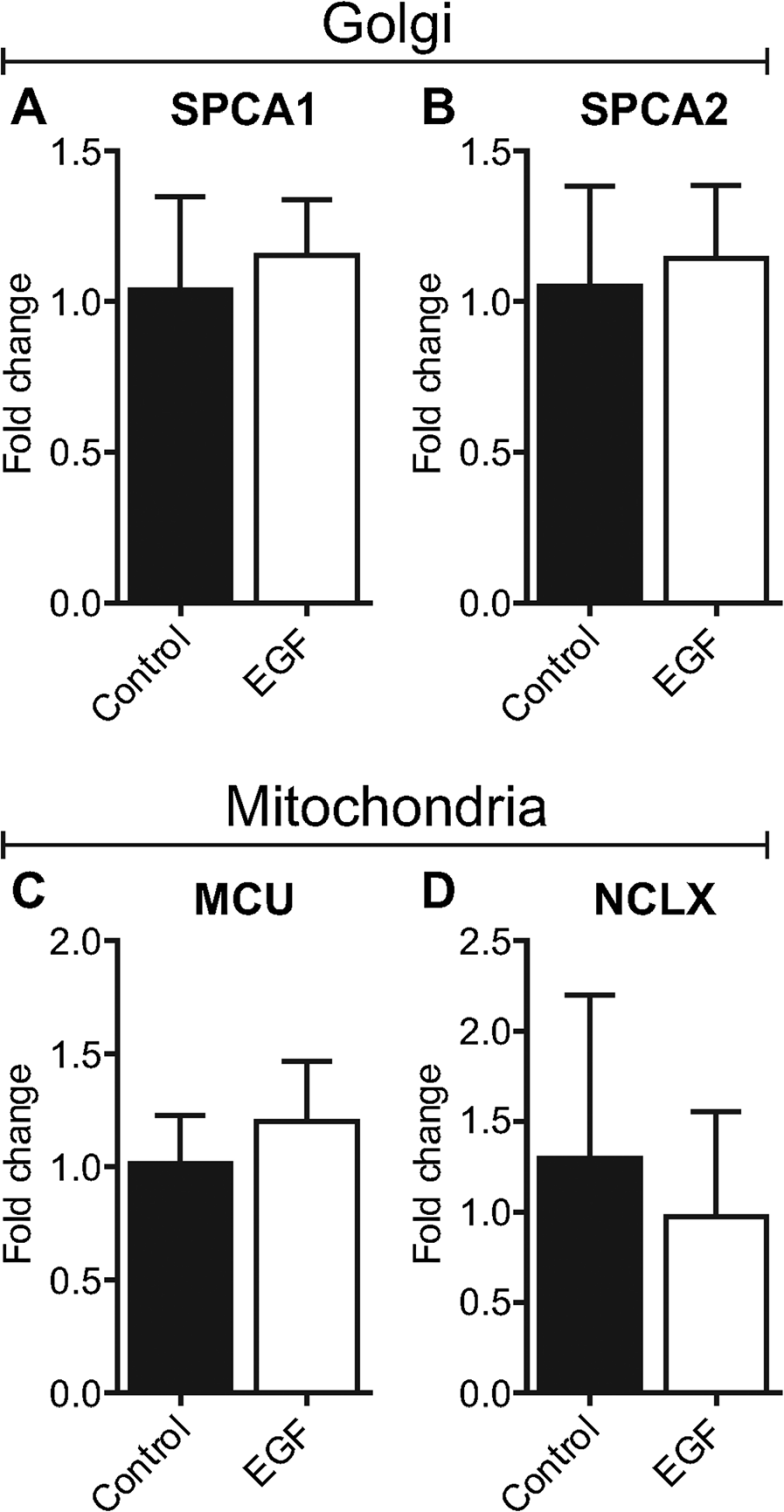
**Assessment of Golgi and mitochondrial calcium transporters with EGF-induced EMT.** Gene expression of **A)** SPCA1, **B)** SPCA2, **C)** MCU and **D)** NCLX with EGF-induced EMT was assessed using real-time RT-PCR. Bar graphs show mean ± S.D. for nine individual wells from three independent experiments. Statistical analysis was performed using an unpaired *t*-test.

SPCAs are regulators of Golgi luminal calcium levels [23]. Recently, we showed that SPCA1 is enriched in basal-like breast cancers, a class of aggressive breast cancers with EMT-like features [24]. Given the potential for SPCAs to regulate processes important for carcinogenesis [24,25] and the observed repositioning of the Golgi, in some cell types, to the leading edge during cell migration [26], we hypothesised that altered Golgi calcium homeostasis may be a feature of EGF-induced EMT. However, no significant alterations in the levels of SPCA1 mRNA (Figure 2A) or its related isoform SPCA2 (Figure 2B) were associated with EGF-induced EMT in MDA-MB-468 breast cancer cells.

### Mitochondria: MCU and NCLX

Excess mitochondrial calcium accumulation is a trigger for cell death [27]. Downregulation of the MCU in HeLa cervical cancer cells by miR-25 protects cells from calcium-dependent apoptosis, and reduced expression of MCU is a feature of poorly differentiated colonic adenocarcinomas [28]. In contrast, MCU expression appears to be enriched in some breast cancer subtypes associated with a poor clinical prognosis [29]. Although several studies have reported that EMT or expression of EMT-associated transcription factors is associated with apoptosis resistance [30]–[32], the mechanism underpinning this process has not been characterised. We evaluated changes in gene expression of two key mitochondrial calcium accumulation and release proteins—the MCU [33,34] and NCLX [35]—in breast cancer cells induced to undergo EMT with EGF. Our results show no significant changes in the expression of the MCU or NCLX with EGF-induced EMT in MDA-MB-468 cells (Figure 2C & D). These findings indicate that if EGF-induced EMT in breast cancer cells is associated with apoptosis-resistance, it is not due to altered gene expression of the MCU or NCLX, at least under these study conditions.

### Endoplasmic reticulum: SERCAs, IP_3_Rs and RYRs

**Figure 3:**
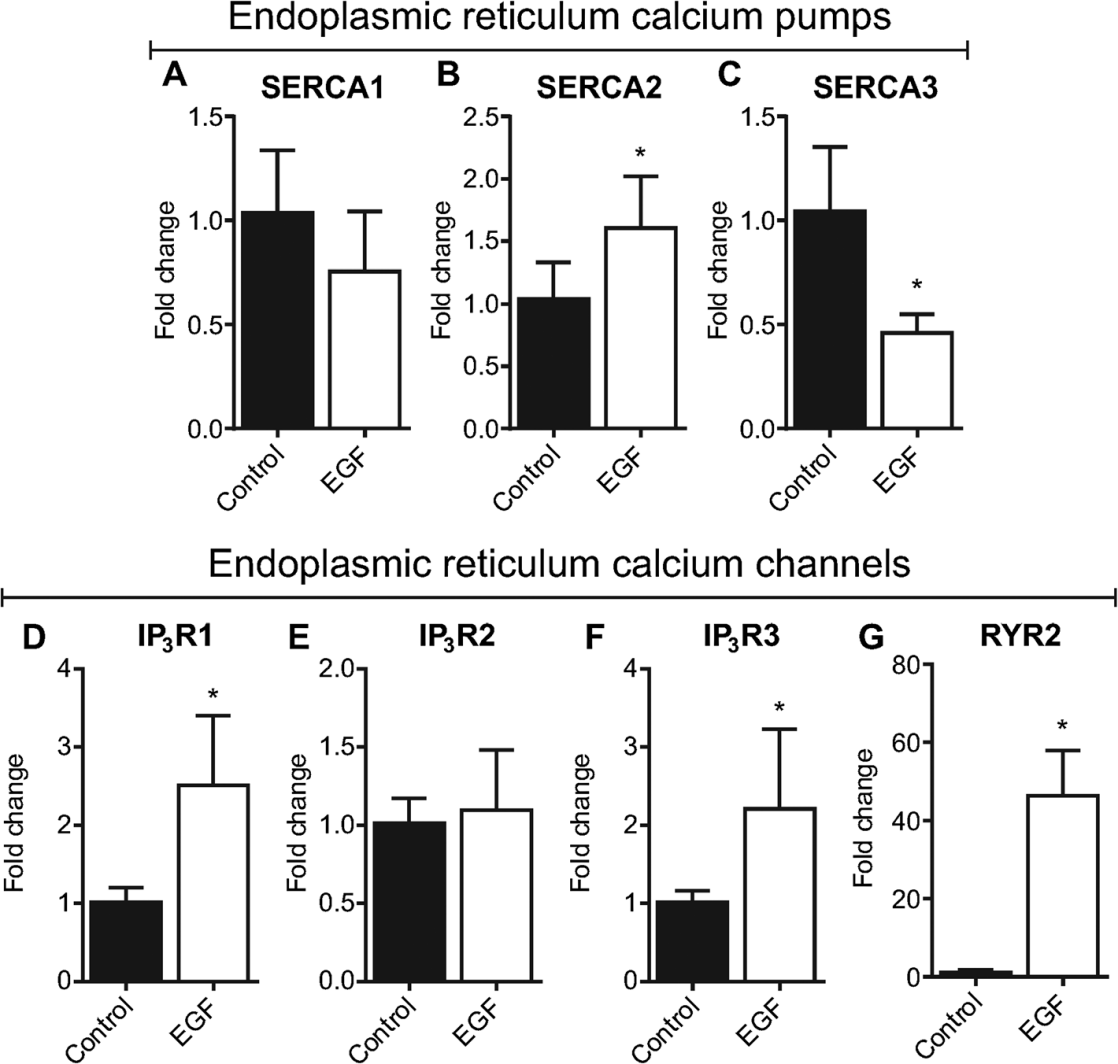
**Assessment of ER calcium channels and pumps with EGF-induced EMT.** Gene expression of **A)** SERCA1, **B)** SERCA2, **C)** SERCA3, **D)** IP_3_R1, **(E)** IP_3_R2, **(F)** IP_3_R3, and **(G)** RYR2 was assessed using real-time RT-PCR. Bar graphs show mean ± S.D. for nine individual wells from three independent experiments. * *P* < 0.05, unpaired *t*-test.

The ER is a major source of releasable calcium in epithelial cells [36]. Luminal ER calcium levels are maintained by SERCA calcium pumps, which are encoded by three genes (ATP2A/SERCA1-3) [37]. Activation of phospholipase C-coupled receptors (e.g., some G-protein coupled receptors) leads to the production of IP_3_ and subsequent activation of the ER calcium-permeable channels IP_3_R1, IP_3_R2 and IP_3_R3 [38]. The nature of the cytosolic calcium signal evoked by IP_3_ is highly dependent on the expression profile of IP_3_Rs [39]. For example, in B cells, activation of IP_3_R2 produces regular and sustained calcium oscillations, while IP_3_R3 generates a calcium response that is characterised by a transient cytosolic calcium peak and few oscillatory spikes [39]. IP_3_R subtypes also differ in their sensitivity to IP_3_[39]. To assess possible alterations in ER calcium handling, we assessed both SERCA and IP_3_R isoform mRNA levels in MDA-MB-468 breast cancer cells induced to undergo EMT. No significant change in SERCA1 gene expression was observed (Figure 3A). However, an increase in SERCA2 and a down-regulation of SERCA3 was associated with EGF-induced EMT (Figure 3B & C). EGF-induced EMT was also associated with a 2.5-fold increase in IP_3_R1 and a 2.2-fold increase in IP_3_R3 mRNA levels (Figure 3 D-F). Changes in gene expression of SERCA2, SERCA3, IP_3_R1 and IP_3_R3 suggest that alterations in ER calcium homeostasis may be a characterising feature of EGF-induced EMT in MDA-MB-468 breast cancer cells.

**Figure 4:**
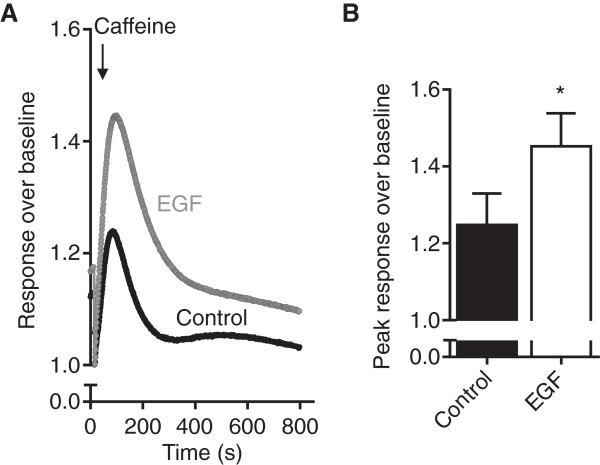
**Cytosolic calcium response to caffeine.** Assessment of cytosolic calcium responses to 10 mM caffeine in MDA-MB-468 breast cells induced to undergo EMT with EGF (24 h). **A)** Average response over baseline and **B)** peak response over baseline. Bar graph shows mean ± S.D for nine individual wells from three independent experiments. * *P* < 0.05, unpaired *t*-test.

To determine whether alterations in ER calcium signalling with EMT could also be mediated via alterations in the ryanodine receptor family of (sarco)-endoplasmic reticulum calcium release channels, mRNA levels of ryanodine receptor isoforms were assessed during EGF-induced EMT. There are three ryanodine receptor isoforms in humans; RYR1 is predominantly expressed skeletal muscle; RYR2 is the abundant isoform in cardiac muscle; and RYR3 expression occurs in various tissues including the brain [40]. RYR1 and RYR3 were not detected in MDA-MB-468 epithelial breast cancer cells and expression of these isoforms was not induced to detectable levels with EGF-induced EMT (data not shown). Levels of mRNA for the RYR2 subtype were low in MDA-MB-468 breast cancer cells prior to EMT induction with EGF. However, EGF-treatment induced a marked (46-fold) increase in levels of RYR2 mRNA (Figure 3G). We were unable to detect RYR2 expression in MDA-MB-468 breast cancer cells with immunoblotting using a commercially available antibody (Abcam C3-33, data not shown); however, corresponding to the increase in RYR2 gene expression, MDA-MB-468 breast cancer cells with a more mesenchymal phenotype exhibited a modest but significantly greater response to the ryanodine receptor activator caffeine, as assessed through caffeine-mediated increases in [Ca^2+^]_CYT_ (Figure 4A & B).

## Conclusions

Alterations in the expression of ER calcium channels and pumps occur in cancer cell lines and clinical cancer samples [12,41]–[45]. For example, expression of SERCA3 is significantly reduced in colon carcinomas and colon cancer cell lines compared to normal colonic epithelia [41], and SERCA3 expression is elevated with chemically-induced differentiation of colon, gastric and lung cancer cell lines [41,42]. Therefore, the reduced SERCA3 mRNA levels observed in this study with EGF-induced EMT may reflect a loss of cellular differentiation congruent with the acquisition of a more stem cell-like phenotype. Future studies are needed to assess whether these changes are related to EMT-associated stemness and to assess the various SERCA transcript variants with EGF-induced EMT. Ryanodine receptor expression, using an antibody that detects all ryanodine receptor isoforms, also correlates with breast cancer tumour grade [43]. Furthermore, the induction of RYR2 expression by factors/conditions known to induce EMT in a variety of cell types (including EGF, TGFβ [46] and hypoxia [47]), suggests that RYR2 may be a key calcium channel for the mesenchymal cell phenotype. The association between RYR2 and EMT requires further investigation.

We have recently shown that the calcium signal is critically required for EMT induction in breast cancer cells [48] and that, in addition to regulating the conversion to a more mesenchymal cell phenotype, EMT is associated with a remodelling of non-stimulated, agonist-stimulated and store-operated calcium influx [6,7]. This study assessed changes in mRNA levels of intracellular calcium channels, pumps and exchangers with EMT. Our results demonstrate that similar to vascular smooth muscle cell phenotypic switching [20], EMT in breast cancer cells is associated with changes in gene expression of specific ER calcium channels and pumps, the consequence of which may be altered ER calcium storage and signalling. This is in agreement with our previously published data showing altered ER calcium release kinetics with EGF-induced EMT in this cell line [6]. Changes in ER calcium handling may lead to altered responses to phospholipase-C-coupled receptor agonists, and may be important for the invasive properties of mesenchymal-like cancer cells.

## Materials and methods

### Cell culture

MDA-MB-468 cells were maintained in Dulbecco’s Modified Eagle’s Medium (D6546, Sigma Aldrich) supplemented with penicillin (100 U/mL, Invitrogen), streptomycin (100 μg/mL, Invitrogen), fetal bovine serum (10%, Sigma Aldrich) and L-glutamine (4 mM, Invitrogen) [6]. To induce EMT, MDA-MB-468 cells were serum-starved (0.5% fetal bovine serum) for 24 h and stimulated with EGF (50 ng/mL, Sigma Aldrich) for 12 h (gene expression) or 24 h (calcium assays) [5]–[7]. These time points were chosen because changes in mRNA levels are expected to precede changes in functional responses. MDA-MB-468 cells were maintained in a humidified incubator at 37°C with 5% CO_2_ and routinely tested negative for mycoplasma infection (MycoAlert, Lonza). Short tandem repeat (STR) profiling of the MDA-MB-468 cell line was performed by the Queensland Institute of Medical Research using the StemElite ID Profiling Kit (Promega).

### RNA isolation and real-time RT-PCR

Total RNA was isolated and purified using the RNeasy Plus Mini Kit (Qiagen). RNA (1000 ng) was reverse transcribed using the Omniscript RT Kit (Qiagen) and cDNA amplified using TaqMan Universal PCR Master Mix or TaqMan FAST Universal PCR Master Mix with a StepOnePlus Real Time PCR System (Applied Biosystems). The following Gene Expression Assays (Applied Biosystems) were used in this study: IP_3_R1 (Hs00181881_m1), IP_3_R2 (Hs00181916_m1), IP_3_R3 (Hs01573555_m1), MCU (Hs00293548_m1), NCLX (Hs00227951_m1), RYR1 (Hs00166991_m1), RYR2 (Hs00892840_m1), RYR3 (Hs00168821_m1), SERCA1 (Hs01092295_m1), SERCA2 (Hs00544877_m1), SERCA3 (Hs00193090_m1), SPCA1 (Hs00205122_m1), SPCA2 (Hs00208296_m1), Twist (Hs00361186_m1) and Vimentin (Hs00185584_m1). Relative quantification was calculated with reference to 18S rRNA and analysed using the comparative C_T_ method [49]. Targets registering above the limit of detection or having C_T_ values greater than 35 were assigned a value of 35.

### FLIPR calcium assays

Measurement of [Ca^2+^]_CYT_ responses to caffeine was performed using a fluorometric imaging plate reader (FLIPR^TETRA^, Molecular Devices) as previously described [24]. Briefly, cells were loaded with Fluo-4 AM (2 μM) in a solution containing PBX Signal Enhancer (5%; PBX No Wash Ca^2+^ Assay Kit, BD Biosciences) and probenecid (500 μM) in physiological salt solution (KCl 5.9 mM, MgCl_2_ 1.4 mM, HEPES 10 mM, NaH_2_PO_4_ 1.2 mM, NaHCO_3_ 5 mM, NaCl 140 mM, glucose 11.5 mM and CaCl_2_ 1.8 mM) [6,50]. Calcium measurements were performed with 470/95 and 515/75 nm excitation and emission filters. Fluorescent values were normalised to the point immediately following agonist addition and are expressed as response over baseline.

### Statistical analysis

Statistical analysis was performed using GraphPad Prism (v6.0 for Windows). Statistical tests used in this study are outlined in each figure legend.

## Abbreviations

[Ca2+]CYT: Cytosolic calcium concentration; CICR: Calcium-induced calcium release; CT: Cycle threshold; EGF: Epidermal growth factor; EMT: Epithelial-mesenchymal transition; ER: Endoplasmic reticulum; FLIPR: Fluorometric imaging plate reader; IP3: Inositol 1,4,5-trisphosphate; IP3R: Inositol 1,4,5-trisphosphate receptor; MCU: Mitochondrial calcium uniporter; NCLX: Na^+^/Ca^2+^ Exchanger; PMCA: Plasma membrane calcium ATPase; RT-PCR: Reverse transcription polymerase chain reaction; RYR: Ryanodine receptor; SERCA: (sarco)-Endoplasmic reticulum calcium ATPase; SPCA: Secretory pathway calcium ATPase; TRP: Transient receptor potential.

## Competing interests

The authors declare that they have no potential conflicts of interest.

## Authors’ contributions

Performed the experiments: FD and MP. Designed or conceived the experiments: FD, MP, GM, SRT and EWT. Contributed reagents/materials/analysis tools: GM and SRT. Wrote the manuscript: FD and GM. Edited the manuscript: SRT, EWT, MP, MOP and PC. All authors read and approved the final manuscript.
